# Perugini Grade 3 Uptake Does Not Exclude AL Cardiac Amyloidosis: When Noninvasive Diagnosis Fails

**DOI:** 10.1016/j.jaccas.2026.109012

**Published:** 2026-09-23

**Authors:** Fulvio Cacciapuoti, Vittorio Taglialatela, Giuseppe Nicoletti, Flavia Casolaro, Massimo Russo, Nicola Verde, Ciro Mauro

**Affiliations:** a Division of Cardiology, “A. Cardarelli” Hospital, Naples, Italy; b Department of Advanced Medical Sciences, “L. Vanvitelli” University, Naples, Italy

**Keywords:** amyloidosis, bone scintigraphy, endomyocardial biopsy, free light chains, HFpEF, monoclonal gammopathy

## Abstract

**Background:**

Bone scintigraphy has improved noninvasive diagnosis of cardiac amyloidosis, but interpretation remains challenging when monoclonal gammopathy is present.

**Case Summary:**

A 76-year-old man with heart failure with preserved ejection fraction, chronic kidney disease, permanent atrial fibrillation, and persistent troponin elevation underwent coronary angiography showing significant left anterior descending artery stenosis treated with percutaneous coronary intervention. Persistent symptoms, recurrent heart failure decompensations, disproportionate natriuretic peptide elevation, increased wall thickness, and apical sparing raised suspicion for infiltrative cardiomyopathy. Laboratory testing revealed monoclonal gammopathy. Tc-99m–pyrophosphate scintigraphy showed Perugini grade 3 uptake, strongly suggestive of transthyretin cardiac amyloidosis (ATTR-CA), but endomyocardial biopsy established light-chain (AL) cardiac amyloidosis. AL-directed therapy was initiated, but the patient died of severe pneumonia with respiratory failure.

**Discussion:**

In the presence of monoclonal gammopathy, even Perugini grade 3 uptake does not establish ATTR-CA. Recent ischemic injury may further confound scintigraphic interpretation.

**Take-Home Message:**

Tissue confirmation remains essential for accurate diagnosis and timely AL-directed therapy.

## Visual Summary

**Figure:**
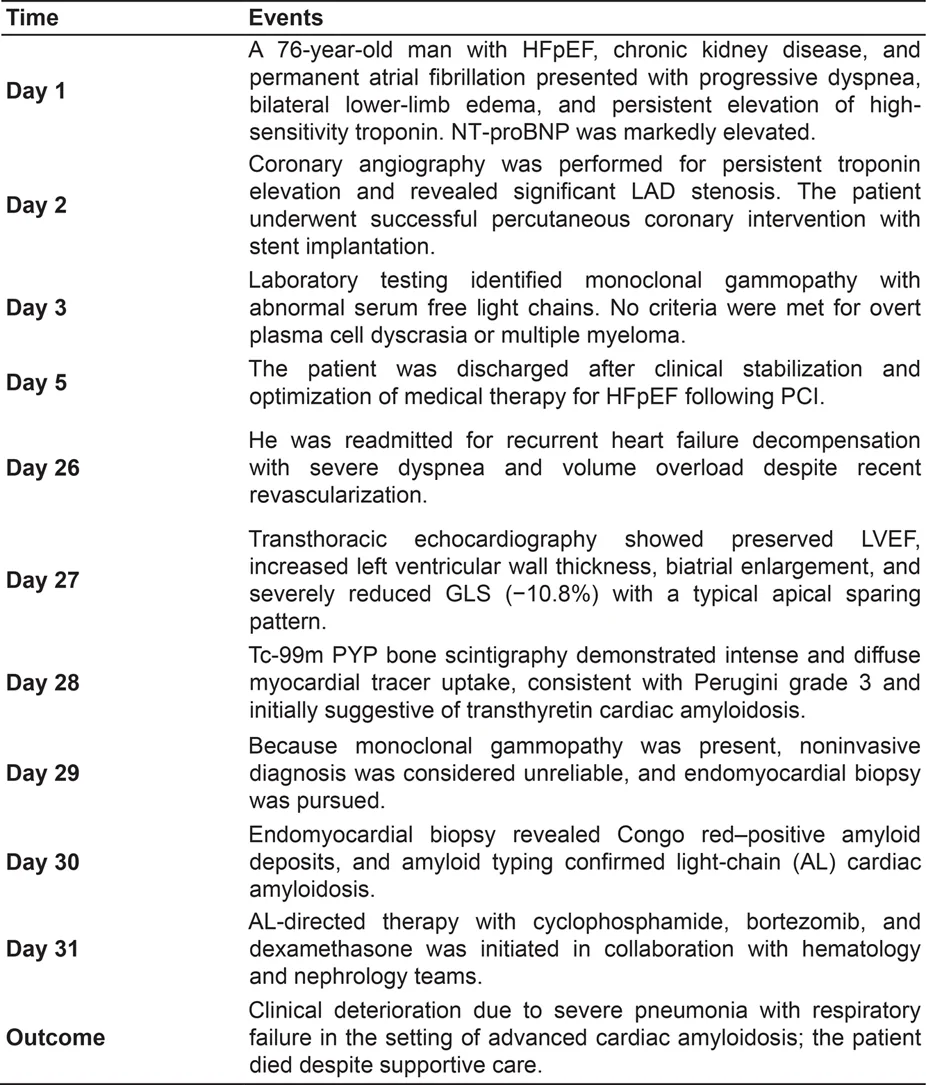

Heart failure with preserved ejection fraction (HFpEF) is a heterogeneous clinical syndrome frequently associated with advanced age and multiple comorbidities. Among its less common but clinically relevant causes, cardiac amyloidosis is increasingly recognized, particularly with the widespread use of advanced imaging techniques.^1^ Bone scintigraphy with technetium-labeled tracers has enabled noninvasive diagnosis of transthyretin cardiac amyloidosis (ATTR-CA)^2^^,^^3^; however, this approach has important limitations. Positive scintigraphy findings have been reported in patients with light-chain (AL) amyloidosis, and the specificity of this technique is significantly reduced in the presence of monoclonal gammopathy.^4^^,^^5^ This case illustrates the diagnostic challenges of cardiac amyloidosis in a patient with HFpEF, coronary artery disease, and monoclonal gammopathy, emphasizing the critical role of endomyocardial biopsy for definitive amyloid typing.

**Take-Home Messages**

• In the presence of monoclonal gammopathy, even Perugini grade 3 myocardial uptake does not establish ATTR-CA and mandates tissue confirmation for accurate amyloid typing.
• In patients with HFpEF, persistent troponin elevation, biomarker disproportion, increased wall thickness, and apical sparing should raise suspicion for cardiac amyloidosis even when competing explanations such as coronary artery disease are present.
• Positive bone scintigraphy should be interpreted cautiously in patients with recent ischemic events, in whom ischemic injury may contribute to tracer uptake and increase the risk of diagnostic misclassification.

## History of Presentation

A 76-year-old man presented with progressive dyspnea and bilateral lower extremity edema. On admission, blood pressure was 105/60 mm Hg, heart rate 75 beats/min with an irregularly irregular rhythm, and oxygen saturation was preserved on room air. Physical examination revealed signs of volume overload without chest pain or fever.

## Past Medical History

The patient's medical history was notable for chronic kidney disease, permanent atrial fibrillation on anticoagulation, HFpEF with multiple prior hospitalizations, and known coronary artery disease. There was no previous diagnosis of hematologic malignancy.

## Differential Diagnosis

Differential diagnoses included ischemic cardiomyopathy related to coronary artery disease, hypertensive heart disease, hypertrophic cardiomyopathy, and infiltrative cardiomyopathies (cardiac amyloidosis and, less likely, sarcoidosis). Given recurrent HFpEF decompensations with biomarker disproportion and a typical apical-sparing pattern on strain imaging, cardiac amyloidosis was prioritized. The presence of monoclonal gammopathy prompted the need for definitive amyloid typing.

## Investigations

The patient had been initially admitted to another tertiary-care center for acute heart failure decompensation associated with persistent elevation of high-sensitivity troponin. Given the persistent troponin elevation and known coronary artery disease, an ischemic etiology was initially prioritized at the referring center. Coronary angiography was performed and revealed a significant stenosis of the left anterior descending artery (LAD), which was successfully treated with percutaneous coronary intervention and stent implantation. Despite revascularization and optimization of medical therapy, the patient continued to experience symptoms of heart failure and was subsequently referred to our center for further evaluation. During hospitalization at our institution, laboratory testing confirmed persistent elevation of cardiac biomarkers and revealed monoclonal gammopathy with abnormal serum-free light chains. Comprehensive hematologic work-up excluded multiple myeloma or other overt plasma cell dyscrasia.

### Interval clinical course

Approximately 3 weeks after discharge from the referring hospital, the patient was admitted to our center because of persistent dyspnea and recurrent heart failure decompensation despite recent coronary revascularization and optimized medical therapy. Given the persistence of symptoms and disproportionate biomarker elevation, an infiltrative cardiomyopathy was suspected.

### Laboratory findings

In light of the clinical presentation, persistent myocardial injury markers, increased ventricular wall thickness, and echocardiographic features suggestive of infiltrative cardiomyopathy, targeted laboratory evaluation was performed, including serum-free light chain assessment. κ free light chains were 43.0 mg/L (reference: 3.3-19.4 mg/L), and λ free light chains 116.0 mg/L (reference: 5.7-26.3 mg/L), with a κ/λ ratio of 0.37, consistent with monoclonal gammopathy. Serum and urine immunofixation confirmed a monoclonal component without criteria for multiple myeloma. At presentation, laboratory findings were consistent with monoclonal gammopathy without criteria for overt plasma cell dyscrasia; definitive AL amyloidosis was established only after tissue confirmation.

### Electrocardiogram

**Figure 1:**
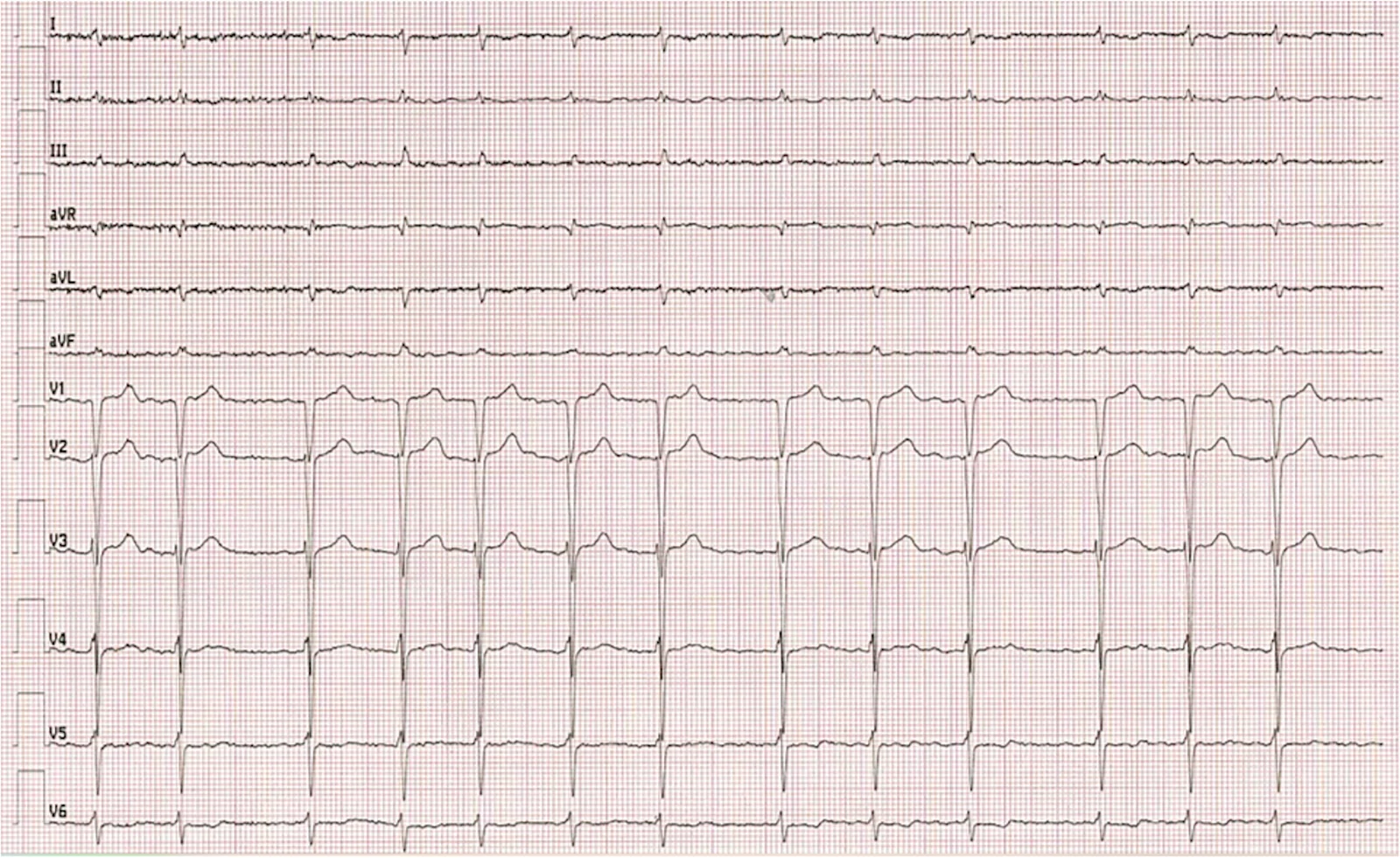
12-Lead ECG Showing Permanent Atrial Fibrillation With Low QRS Voltages in the Precordial Leads Low voltage on surface electrocardiogram, particularly when discordant with increased left ventricular wall thickness on echocardiography, represents a classical red flag for infiltrative cardiomyopathies, including cardiac amyloidosis. In the present case, these electrocardiographic features contributed to raising suspicion for a nonischemic etiology of heart failure despite the presence of concomitant coronary artery disease.

The 12-lead electrocardiogram showed atrial fibrillation with a ventricular rhythm of approximately 70 to 75 beats/min. Low QRS voltages were noted in the precordial leads, along with secondary ST-T changes, without ST-segment elevation or features suggestive of acute myocardial ischemia (Figure 1).

### Transthoracic echocardiography

Transthoracic echocardiography demonstrated a nondilated left ventricle with preserved left ventricular ejection fraction. The ventricular walls appeared increased in thickness and biatrial enlargement. No hemodynamically significant valvular disease was observed, and there was no relevant pericardial effusion.

**Figure 2:**
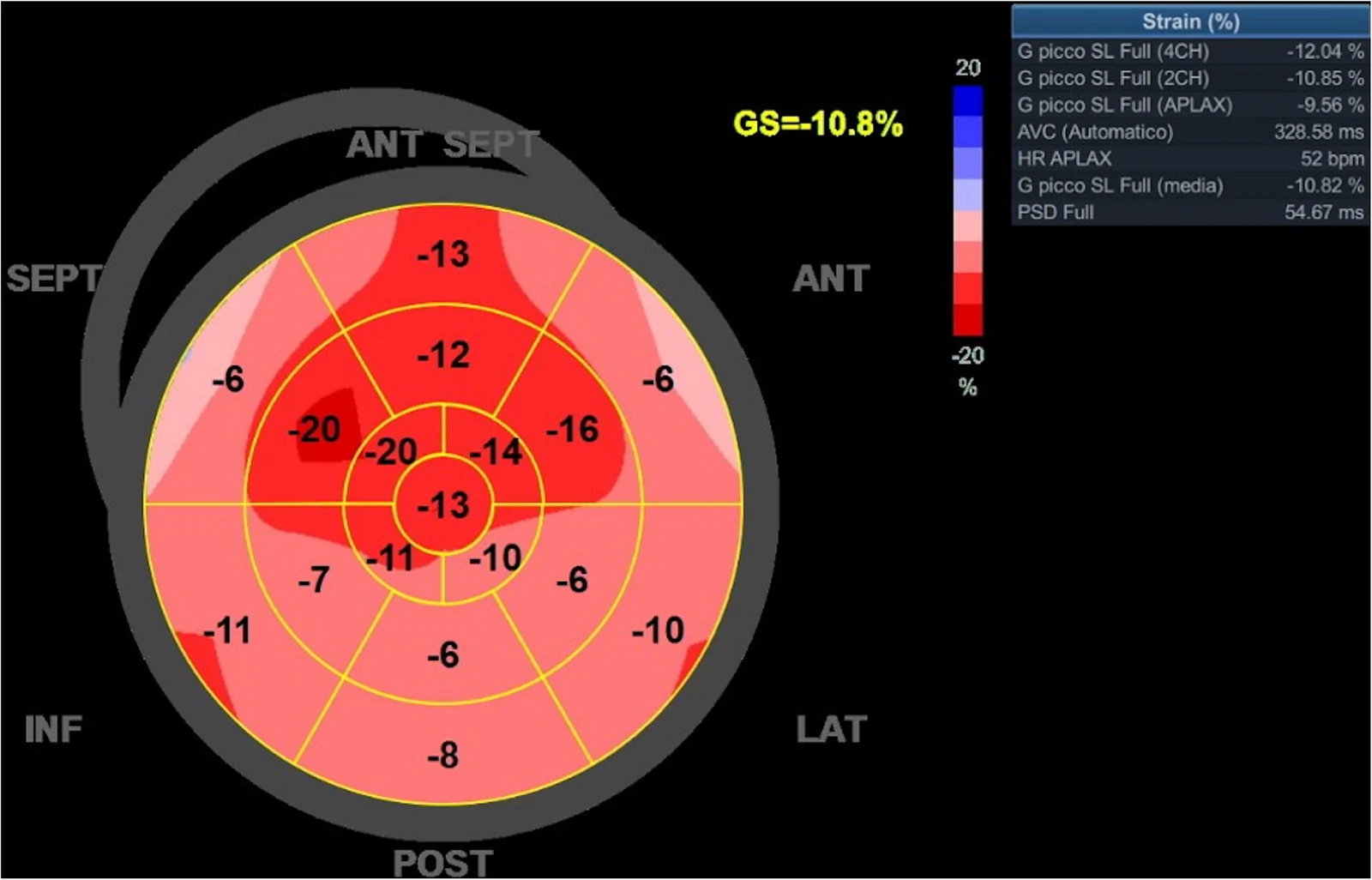
Transthoracic Echocardiography Demonstrated Preserved Left Ventricular Ejection Fraction With Increased Wall Thickness and Biatrial Enlargement Global longitudinal strain (GLS) analysis revealed severely reduced myocardial deformation (−10.8%) with relative preservation of apical segments, producing a characteristic “apical sparing” pattern. This strain distribution is highly suggestive of cardiac amyloidosis and represents an important noninvasive clue in patients with heart failure with preserved ejection fraction and disproportionate biomarker elevation.

Speckle-tracking analysis revealed a severely reduced global longitudinal strain, with a mean value of −10.8%. The corresponding bull's-eye plot showed a characteristic pattern of relative apical sparing, with marked reduction of strain in the basal and midventricular segments (Figure 2).

### Nuclear imaging

**Figure 3:**
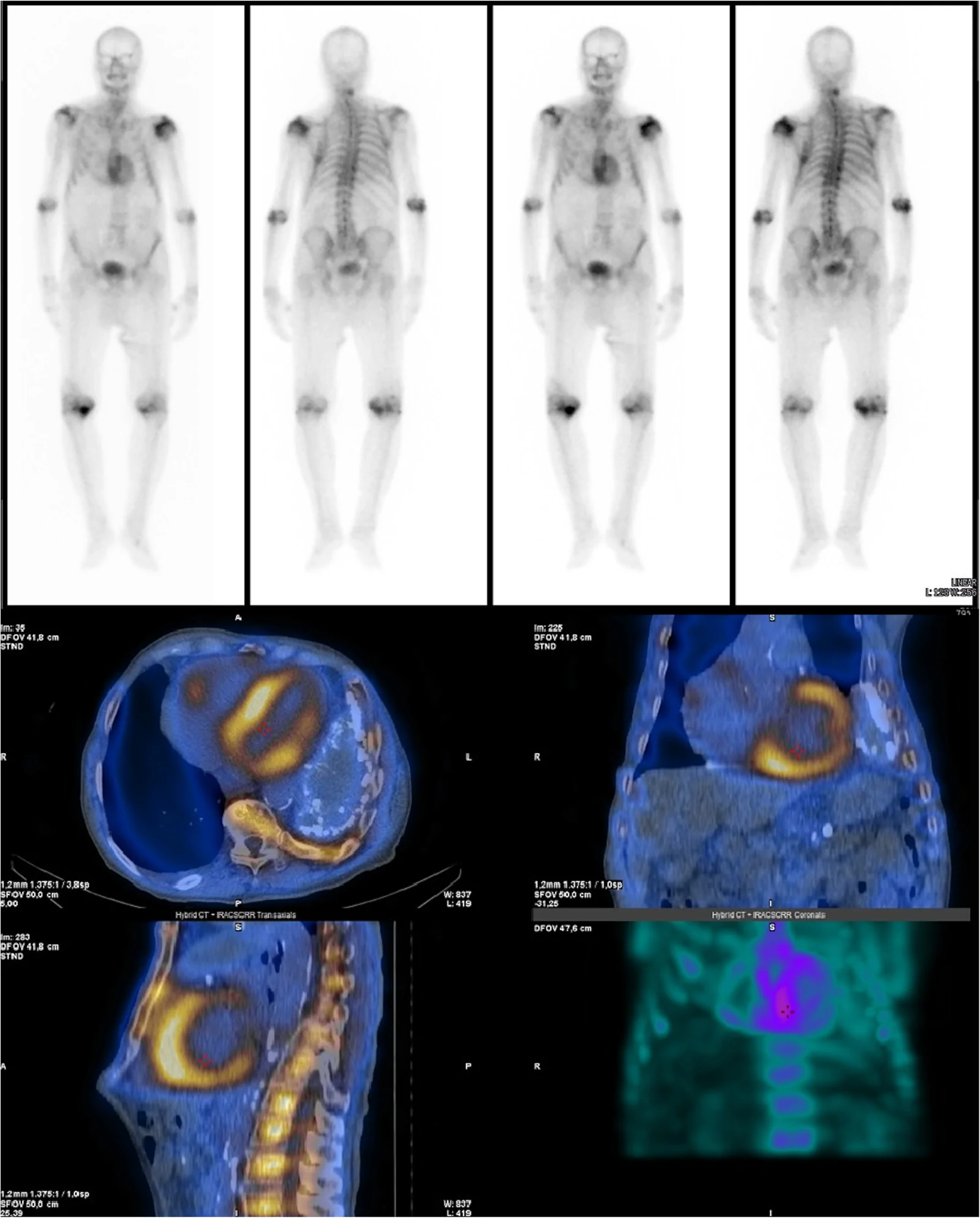
Tc-99m Pyrophosphate Scintigraphy Showing Perugini Grade 3 Myocardial Uptake (Top) Whole-body planar bone scintigraphy demonstrates intense and diffuse myocardial tracer uptake exceeding bone uptake, consistent with Perugini grade 3 myocardial uptake. (Bottom) Hybrid SPECT/CT images (axial, coronal, sagittal, and maximum intensity projection) confirm diffuse myocardial tracer accumulation. SPECT/CT = single-photon emission computed tomography/computed tomography.

To further evaluate the suspicion of infiltrative cardiomyopathy, the patient underwent bone scintigraphy using Tc-99m–pyrophosphate (PYP), with image acquisition performed 3 hours after tracer injection. The heart-to-contralateral lung ratio was 1.82, supporting high-grade myocardial uptake, which would be strongly suggestive of transthyretin cardiac amyloidosis (ATTR-CA) in the absence of monoclonal gammopathy.^2^^,^^6^ Planar and single-photon emission computed tomography (SPECT) images demonstrated intense and diffuse myocardial tracer uptake, exceeding bone uptake, consistent with Perugini grade 3 myocardial involvement (Figure 3).

According to current diagnostic algorithms, this scintigraphic pattern would suggest ATTR-CA only in the absence of monoclonal gammopathy.^2^^,^^3^ However, given the concomitant presence of monoclonal gammopathy, the findings were interpreted with caution, and definitive amyloid typing was deferred pending histologic confirmation. Cardiac magnetic resonance imaging was not pursued because advanced renal dysfunction limited its feasibility and, in the presence of monoclonal gammopathy, tissue confirmation was required regardless of additional imaging findings.

## Management

Despite the scintigraphic findings strongly suggestive of ATTR-CA, the coexistence of monoclonal gammopathy precluded a definitive noninvasive diagnosis. Given the major therapeutic and prognostic implications of amyloid subtype differentiation, the patient was referred for endomyocardial biopsy. Histologic examination revealed interstitial amyloid deposits with Congo red staining and characteristic apple-green birefringence under polarized light. Subsequent amyloid typing by immunohistochemistry and mass spectrometry confirmed AL amyloidosis with cardiac involvement. Following the definitive diagnosis, a bortezomib-based chemotherapy regimen (cyclophosphamide, bortezomib, and dexamethasone) was initiated in close collaboration with hematology, with dose adjustments according to the patient's advanced cardiac involvement and chronic kidney disease. Supportive heart failure therapy was continued, including careful volume management and avoidance of agents often poorly tolerated in cardiac amyloidosis, such as beta-blockers, renin–angiotensin system inhibitors, nondihydropyridine calcium-channel blockers, and digoxin.

## Outcome and Follow-Up

Despite initiation of disease-specific therapy for AL amyloidosis, the patient's clinical course was unfavorable. During follow-up, he developed severe pneumonia, complicated by acute respiratory failure and hemodynamic deterioration in the setting of advanced cardiac involvement. Despite supportive care, his condition progressively worsened, and he died of respiratory failure secondary to severe pneumonia, without clear evidence of predominant cardiogenic shock.

The patient died before a meaningful hematologic response could be achieved, reflecting the advanced stage and aggressive natural history of AL cardiac amyloidosis.

## Discussion

This case illustrates a clinically relevant diagnostic pitfall in cardiac amyloidosis, emphasizing the limitations of noninvasive imaging when monoclonal gammopathy is present. Although abnormal bone scintigraphy in AL amyloidosis is already well documented,^5^ this case retains educational value because even Perugini grade 3 uptake may occur in AL cardiac amyloidosis and be particularly misleading in a clinically confounded setting. The relevance of this report therefore lies less in the rarity of the phenomenon itself and more in the diagnostic pitfall it highlights.

HFpEF is a heterogeneous syndrome, and cardiac amyloidosis remains an underdiagnosed cause. Red flags in this patient included recurrent decompensations, disproportionate NT-proBNP elevation, persistent troponin increase, increased wall thickness, and severely reduced global longitudinal strain with apical sparing. In AL amyloidosis, persistent troponin elevation is a well-recognized feature reflecting ongoing myocardial injury^7^; however, in this case, the coexistence of coronary artery disease and a recent ischemic event further complicates the interpretation of biomarker elevation.

Bone scintigraphy is a powerful diagnostic tool for ATTR-CA when Perugini grade ≥2 uptake is observed in the absence of monoclonal proteins.^2^^,^^3^ However, this algorithm does not apply in patients with monoclonal gammopathy, which is common in elderly HFpEF populations.^5^ In this case, Perugini grade 3 uptake suggested ATTR-CA, but targeted free light chain testing—prompted by the overall clinical and echocardiographic profile—identified monoclonal gammopathy. Endomyocardial biopsy confirmed AL cardiac amyloidosis, demonstrating that AL amyloid may occasionally mimic ATTR-CA on scintigraphy.^8^ Although the identified LAD stenosis was appropriately treated, the lack of clinical improvement suggested that coronary artery disease was not the primary driver of the patient's heart failure syndrome. Reliance on noninvasive imaging alone could have led to diagnostic misclassification and delayed hematologic therapy, particularly detrimental given the aggressive course of AL cardiac amyloidosis. Chronic kidney disease further complicated management and prognosis.

An important limitation of this case is the potential contribution of recent myocardial ischemic injury to the observed scintigraphic findings. Bone-seeking radiotracer uptake is known to occur in areas of myocardial necrosis and may persist for several weeks following an acute myocardial infarction.^9^ Because imaging was performed approximately 3 weeks after coronary revascularization, in the setting of significant LAD disease and troponin elevation, a contribution from recent myocardial injury cannot be excluded. However, the distribution of tracer uptake did not appear to be unequivocally limited to a single coronary territory, although this observation remains interpretative and does not exclude a substantial contribution from recent ischemic injury. Accordingly, a combined contribution of recent myocardial injury and underlying infiltrative disease cannot be excluded. In this context, grade 3 myocardial uptake cannot be considered reliable evidence in favor of ATTR-CA. This further reinforces the need for tissue confirmation whenever monoclonal gammopathy coexists with positive bone scintigraphy.

Existing literature indicates that cardiac tracer uptake on bone scintigraphy is not rare in AL amyloidosis, although it is usually absent, mild, or low grade.^5^^,^^10^^,^^11^ By contrast, high-grade uptake (Perugini grade 2-3), mimicking ATTR-CA, appears to be far less common and has mainly been described in selected series, case reports, and individual false-positive PYP reports.^8^^,^^12^

The mechanisms underlying this phenomenon are not fully understood but may include myocardial damage, microcalcification, and calcium-mediated binding of bone-seeking tracers to damaged myocardial tissue, as well as interactions with amyloid fibrils. In AL amyloidosis, the higher degree of myocardial injury and cellular toxicity associated with light chains may further promote tracer accumulation. These factors may contribute to false-positive imaging findings, even in the absence of transthyretin deposition.

The educational value of this case lies in the convergence of multiple potentially misleading findings: a strongly positive PYP scan, a recent ischemic event that could itself contribute to tracer uptake, and the coexistence of monoclonal gammopathy. Together, these features created a high-risk diagnostic scenario in which reliance on noninvasive assessment alone could have led to inappropriate attribution to ATTR-CA and delay of AL-directed therapy.

## Conclusions

Even Perugini grade 3 myocardial uptake on bone scintigraphy does not establish transthyretin cardiac amyloidosis in the presence of monoclonal gammopathy. Interpretation is particularly challenging in patients with recent ischemic events, in whom ischemic injury may contribute to tracer uptake. In such settings, tissue confirmation remains mandatory to ensure accurate diagnosis, appropriate therapy, and prognostic assessment.

## Funding Support and Author Disclosures

The authors have reported that they have no relationships relevant to the contents of this paper to disclose.

**Visual Summary:** Timeline of Key Clinical Events From Recurrent HFpEF After Coronary Revascularization to the Definitive Diagnosis of Light-Chain Cardiac Amyloidosis Despite Perugini Grade 3 Bone Tracer Uptake

Time	Events
Day 1	A 76-year-old man with HFpEF, chronic kidney disease, and permanent atrial fibrillation presented with progressive dyspnea, bilateral lower limb edema, and persistent elevation of high-sensitivity troponin. NT-proBNP was markedly elevated.
Day 2	Coronary angiography was performed for persistent troponin elevation and revealed significant LAD stenosis. The patient underwent successful PCI with stent implantation.
Day 3	Laboratory testing identified monoclonal gammopathy with abnormal serum-free light chains. No criteria were met for overt plasma cell dyscrasia or multiple myeloma.
Day 5	The patient was discharged after clinical stabilization and optimization of medical therapy for HFpEF following PCI.
Day 26	He was readmitted for recurrent heart failure decompensation with severe dyspnea and volume overload despite recent revascularization.
Day 27	Transthoracic echocardiography showed preserved LVEF, increased left ventricular wall thickness, biatrial enlargement, and severely reduced GLS (−10.8%) with a typical apical sparing pattern.
Day 28	Tc-99m-PYP bone scintigraphy demonstrated intense and diffuse myocardial tracer uptake, consistent with Perugini grade 3 and initially suggestive of transthyretin cardiac amyloidosis.
Day 29	Because monoclonal gammopathy was present, noninvasive diagnosis was considered unreliable, and endomyocardial biopsy was pursued.
Day 30	Endomyocardial biopsy revealed Congo red–positive amyloid deposits, and amyloid typing confirmed AL cardiac amyloidosis.
Day 31	AL-directed therapy with cyclophosphamide, bortezomib, and dexamethasone was initiated in collaboration with hematology and nephrology teams.
Outcome	Clinical deterioration due to severe pneumonia with respiratory failure in the setting of advanced cardiac amyloidosis; the patient died despite supportive care.

AL = light-chain amyloidosis; HFpEF = heart failure with preserved ejection fraction; GLS = global longitudinal strain; LAD = left anterior descending artery; LVEF = left ventricular ejection fraction; NT-proBNP = N-terminal pro–B-type natriuretic peptide; PCI = percutaneous coronary intervention; PYP = pyrophosphate.
